# Relative contribution of essential and non-essential activities to SARS-CoV-2 transmission following the lifting of public health restrictions in England and Wales

**DOI:** 10.1017/S0950268822001832

**Published:** 2022-12-07

**Authors:** Susan Hoskins, Sarah Beale, Vincent Nguyen, Yamina Boukari, Alexei Yavlinsky, Jana Kovar, Thomas Byrne, Ellen Fragaszy, Wing Lam Erica Fong, Cyril Geismar, Parth Patel, Annalan M. D. Navaratnam, Martie van Tongeren, Anne M. Johnson, Robert W. Aldridge, Andrew Hayward

**Affiliations:** 1 Centre for Public Health Data Science, Institute of Health Informatics, University College London, London, NW1 2DA, UK; 2 Institute of Epidemiology and Health Care, University College London, London, WC1E 7HB, UK; 3 Department of Infectious Disease Epidemiology, London School of Hygiene and Tropical Medicine, Keppel Street, London, WC1E 7HT, UK; 4 Centre for Occupational and Environmental Health, School of Health Sciences, Faculty of Biology, Medicine and Health, University of Manchester, Manchester, Greater Manchester, UK; 5 Institute for Global Health, University College London, London, WC1N 1EH, UK

**Keywords:** Coronavirus, COVID-19, infectious disease epidemiology, public health, respiratory infections

## Abstract

**Purpose:**

We aimed to understand which non-household activities increased infection odds and contributed greatest to SARS-CoV-2 infections following the lifting of public health restrictions in England and Wales.

**Procedures:**

We undertook multivariable logistic regressions assessing the contribution to infections of activities reported by adult Virus Watch Community Cohort Study participants. We calculated adjusted weighted population attributable fractions (aPAF) estimating which activity contributed greatest to infections.

**Findings:**

Among 11 413 participants (493 infections), infection was associated with: leaving home for work (aOR 1.35 (1.11–1.64), aPAF 17%), public transport (aOR 1.27 (1.04–1.57), aPAF 12%), shopping once (aOR 1.83 (1.36–2.45)) *vs.* more than three times a week, indoor leisure (aOR 1.24 (1.02–1.51), aPAF 10%) and indoor hospitality (aOR 1.21 (0.98–1.48), aPAF 7%). We found no association for outdoor hospitality (1.14 (0.94–1.39), aPAF 5%) or outdoor leisure (1.14 (0.82–1.59), aPAF 1%).

**Conclusion:**

Essential activities (work and public transport) carried the greatest risk and were the dominant contributors to infections. Non-essential indoor activities (hospitality and leisure) increased risk but contributed less. Outdoor activities carried no statistical risk and contributed to fewer infections. As countries aim to ‘live with COVID’, mitigating transmission in essential and indoor venues becomes increasingly relevant.

## Introduction

The COVID-19 pandemic has led to levels of hospitalisation and mortality that are unprecedented in recent history and in March 2020 the UK Government imposed a range of non-pharmaceutical interventions (NPIs) with the aim of minimising transmission of severe acute respiratory syndrome coronavirus 2 (SARS-CoV-2) [1]. These NPIs encompassed strict impositions made on social mixing through a series of ‘lockdowns’ including advice to work from home where possible, closure of non-essential businesses such as hospitality and leisure venues and restrictions on social gatherings. During periods of intense control essential shops remained open and essential workers were able to leave home for work. After 16 months of varying degrees of local and national restrictions, on 19 July 2021, in the fourth step of the national roadmap for easing restrictions in England and Wales, the majority of restrictions were lifted nationally, allowing, amongst other new liberties, people to return to hospitality and leisure venues and to attend social gatherings of unrestricted numbers [2]. The day became colloquially known as ‘Freedom Day’ [3]. Yet, with the continued presence of highly transmissible variants, it is essential to understand which settings contribute the greatest to infections in order to develop targeted mitigation strategies.

Whilst transmission can take place in any setting, the relative importance of different non-household settings and activities to the spread of infection is influenced by the amount of transmission that occurs in different settings and on restrictions and mitigation measures in place at the time [4]. During periods of tight restrictions in England and Wales, occupation was identified as an important predictor of SARS-CoV-2 infection and mortality risk, with a high proportion of the differential risk between occupations likely to be related to differential ability to work from home during periods of intense SARS-CoV-2 transmission [5, 6]. During lockdown, activities which increase social-mixing increased the odds of SARS-CoV-2 acquisition and contributed greatest to non-household acquired infections: leaving home for work or education (adjusted odds ratio (aOR) 1.20 (1.02–1.42), adjusted population attributable fraction (aPAF) 6.9%); public transport (more than once per week aOR 1.82 (1.49–2.23), public transport aPAF 12.42%); and shopping (more than once per week aOR 1.69 (1.29–2.21), shopping aPAF 34.56%) [7, 8]. Other non-household activities were rare during the period under restrictions which closed most pubs, restaurants, bars, club, theatres, cinemas and concert venues, and these activities were not significantly associated with infection [7, 8].

It is clear that the importance of different venues such as hospitality, retail and leisure on population infection rates depends on both the likelihood of transmission occurring within a particular environment and the frequency with which people visit that setting, but the relative importance of these settings has been difficult to assess during periods of intense control measures [9]. We aimed to understand the relative importance of different activities and settings in the transmission of SARS-COV-2 in England and Wales in the period following the lifting of the majority of national public health restrictions on ‘Freedom Day’, on 19 July 2021.

## Methods

The analyses are based on the Virus Watch Cohort, a study approved by the Hampstead NHS Health Research Authority Ethics Committee. Ethics approval number – 20/HRA/2320 and the detailed methodology of which is described elsewhere [10]. Briefly, the study recruits whole households with detailed baseline information, weekly surveys of symptoms and self-reporting of positive SARS-COV-2 tests (PCR or lateral flow) conducted through the national tracing programme, linkage to the national testing dataset and monthly questionnaires on contact and activity patterns.

### Study participants

Within the Virus Watch community cohort study in England and Wales, we identified a cohort of adult participants aged 18 and above who completed three monthly behavioural surveys following the declaration of ‘Freedom Day’ when most restrictions had been lifted (completed during the periods 22/09/2021–29/09/2021, 19/10/2021–26/10/2021 and 16/11/2021–23/11/2021) and cases were included if testing PCR or lateral flow positive between 01/09/2021 and 16/12/2021 (before the widespread circulation of the Omicron variant), unless there was evidence of recent infection in the previous 3 months. We did not include responses from the August survey as many participants were on holiday and survey completion rates were low.

### Outcome variable

SARS-CoV-2 infection was defined as having any of (i) a positive self-reported PCR test, (ii) a positive self-reported lateral flow test, (iii) a positive PCR or lateral flow test from data linkage to the National Testing data. The primary source of data was the Virus Watch dataset. To maximise data on cases, we linked to Public Health England's Second Generation Surveillance System (SGSS), whose ‘testing pillars’ contain national records of SARS-CoV-2 results on current infections, using data from hospitalisations (pillar 1) and community testing (pillar 2) [11]. Linkage was conducted by NHS Digital. There was a high degree of overlap between participant self-report and the SGSS linkage; however, SGSS linkage was used as the preferred source due to potential for greater accuracy regarding dates.

### Exposure variables

Survey respondents reported the number of days they undertook a range of activities during each week in monthly surveys. From the monthly surveys, we examined the frequency of leaving home to go to work or education, using public or shared transport, going to retail settings, visiting indoor and outdoor hospitality or leisure settings in the week prior to the survey. Participants also provided information on the number of known close contacts outside the household during the week before each survey, where close contact was defined as being within 2 m of someone for more than 15 min.

### Statistical methods

**Fig. 1.**
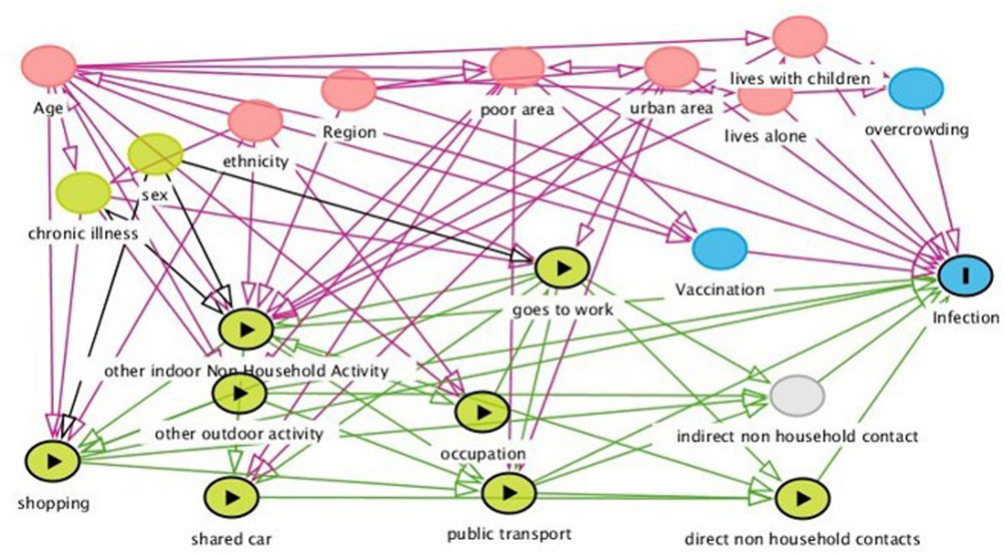
Directed acyclic graph illustrating the causal associations between different activity exposures, covariables and infection.

The weekly frequency of individual activities and number of close contacts was averaged over the three monthly surveys to give an average weekly frequency of activities and average number of contacts, as a proxy for activity patterns during the period post-Freedom Day. We created composite variables for public transport activities (combining use of taxi, bus, over and underground rail and air travel), retail activities (combining use of essential and non-essential shops) and indoor hospitality (eating in an indoor restaurant, cafe or canteen; going to an indoor bar, pub or club; and going to an indoor party), outdoor hospitality (eating in an outdoor restaurant, cafe or canteen; going to an outdoor bar, pub or club; and going to an outdoor party), indoor leisure (attending a gym, the theatre, the cinema, a concert or sports event), outdoor leisure (outdoor team sport), non-social activities (visiting barber, hairdresser, beautician or nail salon). We undertook univariate analyses comparing the proportion with evidence of infection according to weekly frequency of going to work, the composite measures and each exposure individually. We used a directed acyclic graph (DAG) to determine which demographic and other variables to adjust the main activity exposures in multivariate logistic regression modelling (Fig. 1). Based on our DAG the following adjustment set was required to provide with minimal bias an estimate of the direct effect of each non-household activity exposure: mutual adjustment for each activity, region, vaccine status, living alone, living with children and living in a deprived area (using a combination of rural, urban, conurbation area of residence and deprivation status). The DAG suggested two models and the results of the second (which adjusted for ethnicity in place of vaccination status but held all other variables constant) are contained in the Supplementary materials for comparison. Additional variables listed in Figure 1 were not adjusted for due to being either on the causal pathway or to prevent overadjustment. We hypothesised that the risk of infection acquired outside the household is strongly associated with the number of known close contacts outside the household, an effect we have previously observed [8], and as such might impact observed effects particularly for going to work. Close contacts may be on the causal pathway between activities and infection so we therefore conducted a sensitivity analysis additionally adjusting for number of known contacts outside the home, in order to identify settings that remained significantly associated with infection after controlling for this. By doing this we potentially gain a better idea of how some settings may be more influenced than others by transmission from unrecognised close contact, as opposed to recognised close contact. Additionally, while age was not identified through the DAG in our analysis plan, we conducted a further sensitivity analysis adjusting for age, due to the effect of age being a classical confounder for the behavioural activities. Due to the older age structure of our Virus Watch cohort regularly answering the monthly surveys during these periods, we used inverse probability weighting to calculate age-weighted fully adjusted multivariate population attributable fractions (aPAF) (the proportion of non-household transmission in the cohort thought to be attributable to each exposure), with estimates of the UK population age structure taken from the ONS [12]. Missing data were sparse and while included in the univariate analyses, participants with missing data were not included in the multivariate adjusted models and do not contribute to the population attributable fractions (PAFs). Analyses were carried out using STATA version 16.

## Results

### Activities associated with increased infection

During the period (1 September to 16 December 2021), among 11 413 participants, 493 cases were identified (Table 1). The cohort was made up of 57% females, and nearly half (47%) were of working age (18–64 years). The majority (94%) of participants had received at least one dose of a COVID-19 vaccine at entry to this study period, and most (91%) were of White British ethnicity. The majority (75%) of participants lived with another person and few (6%) lived with children. Just under half of participants (46%) lived in an urban area and most participants (58%) lived in postcodes classified as low deprivation according to the UK Office for National Statistics (Table 1).

**Table 1.** Risk of infection among Virus Watch participants

Characteristic	Category	*N* = 11 413 (% in category)	Number of infections *n* = 493 (% within category)	Unadjusted odds ratio	95% CI	*P*
Sex	Male	4857 (43%)	209 (4.3%)	1.00		0.9869
	Female	6470 (57%)	278 (4.3%)	0.99	0.83–1.19	
	Missing	86				
Age	Working age	5370 (47%)	331 (6.2%)	2.38	1.97–2.89	<0.0001
	65 and above	6042 (53%)	162 (2.7%)	1.00		
	Missing	1				
Vaccine status at study entry	Yes	10 726 (94%)	464 (4.2%)	1.00		0.8955
	No	687 (6%)	29 (4.3%)	0.97	0.66–1.43	
Ethnic group	White British	10 286 (91%)	433 (4.2%)	1.00		0.1882
	White other	628 (6%)	31 (4.9%)	1.18	0.81–1.72	
	Asian	213 (2%)	9 (4.2%)	1.00	0.51–1.97	
	Black	54 (<1%)	5 (9.3%)	2.32	0.92–5.86	
	Mixed	91 (1%)	8 (8.8%)	2.19	1.05–4.56	
	Other	49 (<1%)	1 (2.0%)	0.47	0.07–3.44	
	Missing	92				
Region	East Midlands	1065 (9%)	35 (3.3%)	1.00		0.0028
	East of England	2551 (23%)	92 (3.6%)	1.10	0.74–1.64	
	London	1202 (11%)	57 (4.7%)	1.47	0.95–2.25	
	North East	581 (5%)	35 (6.0%)	1.89	1.17–3.05	
	North West	1192 (11%)	62 (5.2%)	1.61	1.06–2.46	
	South East	2210 (19%)	102 (4.6%)	1.42	0.96–2.11	
	South West	924 (8%)	33 (3.6%)	1.09	0.67–1.77	
	Wales	266 (2%)	18 (6.8%)	2.14	1.19–3.83	
	W. Midlands	626 (6%)	33 (5.3%)	1.64	1.01–2.66	
	Yorkshire and The Humber	615 (5%)	15 (2.4%)	0.74	0.39–1.36	
	Missing	181				
Lives alone	Lives alone	2869 (25%)	81 (2.8%)	1.00		
	Lives with someone	8544 (75%)	412 (4.8%)	1.74	1.37–2.22	<0.0001
Lives with children	No	10 679 (94%)	404 (3.8%)	1.00		<0.0001
	Yes	734 (6%)	89 (12.1%)	3.51	2.57– 4.47	
Residential area	Any rural	2886 (26%)	106 (3.7%)	1.00		0.0201
	Any urban	5209 (46%)	216 (4.2%)	1.13	0.89–1.43	
	Any conurbation	3137 (28%)	160 (5.1%)	1.41	1.09–1.81	
	Missing	181				
Deprivation score (IMD quintile) 1 = most deprived	1	852 (8%)	44 (5.2%)	1.36	0.96–1.92	0.3764
	2	1589 (14%)	69 (4.3%)	1.13	0.84–1.52	
	3	2290 (20%)	94 (4.1%)	1.07	0.81–1.39	
	4	3032 (27%)	141 (4.7%)	1.21	0.95–1.55	
	5	3469 (31%)	134 (3.9%)	1.00		
	Missing	181				

There was strong evidence that infection with SARS-CoV-2 was associated with leaving home to go to work or education (OR 1.64 (1.37–1.96)) and this association remained strong after multivariable adjustments (aOR 1.35 (1.11–1.64)) (Table 2). There was strong evidence that infection was independently associated with public transport use (aOR 1.27 (1.04–1.57)). For shopping there were very few participants who reported no shopping, and the greatest odds of infection was observed among those who used shops up to once a week (aOR 1.83 (1.36–2.45)) compared to those who shopped more than three times a week, with increasing frequency reducing the odds of infection (aOR for between one and three times a week 1.51 (1.19–1.92)). There was good evidence that indoor hospitality (aOR 1.21 (0.98–1.48)) and indoor leisure (aOR 1.24 (1.02–1.51)) activities were associated with increased odds of infection, while we found no strong evidence that outdoor hospitality (aOR 1.14 (0.94–1.39)) or outdoor leisure activities (aOR 1.14 (0.82–1.59)) were associated with increased odds of infection. We found no evidence of increased risk of infection for non-social personal care activities such as attending a barber, hairdresser or beautician (aOR 0.74 (0.59–0.90)), the results suggesting a counter-intuitive protecting effect. The observed effect of leaving home to go to work was greatly ameliorated when adjusting for age (aOR 1.09 (0.88–1.33)), but there were no major changes in direction of effect or significance for the remaining activities having adjusted for age (Supplementary Table S1).

**Table 2.** Infection by composite measures, unadjusted and adjusted for region, vaccine status, living alone, living with children, living in a deprived area

Activity	Category	*N* = 11 413 (% in category)	Number of infections *n* = 493 (% within category)	Unadjusted odds ratio (95% CI), *P*	Adjusted odds ratio (95% CI), *P* (*n* = 11 232)	Population attributable fractions (weighted)
Leaves home for work or education	None	7294 (64%)	259 (3.6%)	1.00	1.00	16.5 (5.6–26.2)
	At least once	4119 (36%)	234 (5.7%)	1.64 (1.37–1.96) *P* < 0.0001	1.35 (1.11–1.64) *P* = 0.0029	
All public transport (no car share)	None	5902 (52%)	219 (3.7%)	1.00	1.00	12.2 (1.6–21.6)
	Any	5511 (48%)	274 (4.9%)	1.36 (1.13–1.62) *P* = 0.0009	1.27 (1.04–1.57) *P* = 0.0211	
Retail	None	482 (4%)	14 (2.9%)	0.85 (0.49–1.50)	1.05 (0.58–1.88)	*Not estimated
	Up to once	2022 (18%)	110 (5.4%)	1.64 (1.25–2.16)	1.83 (1.36–2.45)	
	More than once to three times	5745 (50%)	262 (4.6%)	1.37 (1.09–1.72)	1.51 (1.19–1.92)	
	More than three times	3164 (28%)	107 (3.4%)	1.00 *P* = 0.0010	1.00 *P* = 0.0001	
Indoor hospitality and socialising (pub, restaurant, party)	Up to once	6531 (57%)	258 (3.9%)	1.00	1.00	7.3 (−0.9 to 14.8)
	More than once	4882 (43%)	235 (4.8%)	1.23 (1.03–1.47) *P* = 0.0254	1.21 (0.98–1.48) *P* = 0.0710	
Outdoor hospitality and socialising (pub, restaurant, party)	None	6777 (59%)	276 (4.1%)	1.00	1.00	4.7 (−2.5 to 11.4)
	At least once	4636 (41%)	217 (4.7%)	1.16 (0.96–1.39) *P* = 0.1179	1.14 (0.94–1.39) *P* = 0.1860	
Indoor leisure (gym, theatre/cinema, etc.)	None	6713 (59%)	258 (3.8%)	1.00	1.00	9.6 (0.7–17.6)
	At least once	4700 (41%)	235 (5%)	1.32 (1.01–1.58) *P* = 0.0029	1.24 (1.02–1.51) *P* = 0.0284	
Outdoor leisure (team sport outdoors)	None	10 570 (93%)	449 (4.3%)	1.00	1.00	1.14 (−1.9 to 4.2)
	At least once	843 (7%)	44 (5.2%)	1.24 (0.90–1.71) *P* = 0.1943	1.14 (0.82–1.59) *P* = 0.4521	
Non-social activity (hairdresser/beautician, etc.)	None	7469 (65%)	351 (4.7%)	1.00	1.00	−8.7 (−14.4 to −3.3)
	At least once	3944 (35%)	142 (3.6%)	0.76 (0.62–0.92) *P* = 0.0053	0.74 (0.59–0.90) *P* = 0.0031	

The risk of infection increased with an increasing number of known contacts outside the home (for more than five contacts OR 1.21 (1.01–1.45)), although this association did not remain after multivariable adjustments for non-household activities and demographics (aOR 1.05 (0.86–1.28)) (Supplementary Table S1). Supplementary Table S1 shows the effect of leaving home for work or education, public transport use, shopping, indoor and outdoor hospitality and leisure and non-social activities after additionally controlling for the number of close contacts outside the home. The AORs were minimally affected by adding the number of non-household close contacts as a covariate.

For specific public transport activities (Supplementary Table S2), after the demographic adjustments for region, vaccine status, living alone, living with children, residential area and deprivation, there was strong evidence that using a taxi was associated with an increased odds of infection (aOR 1.28 (1.03–1.59)), as was using a bus (aOR 1.27 (1.03–1.56)) and using an overground train or tram (aOR 1.41 (1.14–1.74)). We found no evidence of increased odds with shared car use (OR 0.99 (0.83–1.20)), underground (OR 1.14 (0.87–1.51)) or using an airplane (OR 1.04 (0.76–1.41)).

For other hospitality and leisure activities, after the demographic adjustments, we found strong evidence that all indoor hospitality and leisure activities were associated with an increased risk of infection (Supplementary Table S3): going to an indoor pub, bar or club more than once per week (aOR 1.45 (1.13–1.84)), eating at an indoor restaurant, café or canteen up to once a week (aOR 1.38 (1.11–1.73)), going to an indoor party (aOR 1.38 (1.08–1.77)), going to the gym or indoor sports (OR 1.32 (1.07–1.63)) and going to the theatre, cinema, concert or sports event (aOR 1.23 (1.01–1.49)). We found little or no increased risk of infection for outdoor hospitality and leisure activities (Table S3): going to an outdoor pub, bar or club (aOR 1.09 (0.87–1.36)), eating at an outdoor restaurant, café or canteen (aOR 1.14 (0.94–1.39)), going to an outdoor party (aOR 0.91 (0.55–1.49)) or playing a team sport outdoors (aOR 1.23 (0.89–1.71)).

The AORs for the activities were minimally affected in the sensitivity analyses using the adjustment strategies proposed by the alternative DAG (Supplementary Table S4).

### Relative contribution of specific activities to non-household acquired infections

Given the inverse relationship between shopping frequency and infection (Table 2) we did not calculate PAF for shopping. Amongst other activities, leaving home for work accounted for the greatest contribution to infections (aPAF 17%), followed by public transport use (aPAF 12%), indoor leisure (aPAF 10%), indoor hospitality (aPAF 7%), outdoor hospitality (aPAF 5%) and outdoor leisure (aPAF 1%).

## Discussion

Undertaking essential activities such as leaving home for work (aOR 1.35 (1.11–1.64), APAF 17%) and using public transport (aOR 1.27 (1.04–1.57), APAF 12%) carried the greatest infection risk and were the dominant contributors to SARS-CoV-2 infections in the post-Freedom Day period. Although those going to work had a substantially higher risk of infection, the effect was greatly ameliorated when adjusting for age (1.09 (0.88–1.33)) (Table S1), suggesting that part of the risk associated with going to work is related to the age of those going. This significant decrease after adjusting for age may be explained by the Virus Watch participants who routinely completed the surveys during the relevant months, the majority of whom were of retirement age (53%) and two-thirds of whom (64%) never left home for work. PAFs are influenced by the frequency of exposures within the cohort, and if our cohort leaves home to attend work less often than average this will lead to underestimation of the relevant PAF. We sought to minimise this bias by weighting the PAFs to the national age structure of the population. At times when the risk of infection associated with working outside the home is high, and the associated risk of infection from needing to use public transport to get to work remains, policies supporting at-home working will assist in lowering transmission of respiratory viruses. Office work may not return to pre-pandemic patterns due to a proportion of eligible workers electing to work partly or fully remotely, thereby reducing their individual risk. However, for those who have less choice about the ability to work from home, particularly those reliant on public transport for commuting, policies that mitigate infection risk during essential outside-the-home-work, as well as financial and practical support to limit exposure during periods of intense transmission may reduce inequalities of individual exposure risk.

Despite the lifting of the majority of restrictions and an increasing engagement in social activities, non-essential activities which increased risk (indoor hospitality (aOR 1.21, APAF 7%), indoor leisure activities (aOR 1.24, APAF 10%)), at a level similar to public transport use, did not account for a high proportion of infections. A slightly smaller proportion of participants undertook indoor hospitality (43%) and leisure activities (41%) compared to public transport use (48%) and as frequency of exposure influences estimation of PAFs, this will have accounted, in part, for their smaller contribution to overall infections. The proportion of Virus Watch participants leaving home for work (36%) post-Freedom Day and the contributory role of working outside the home to infections (aPAF 17%) is similar to that observed in the period under national restrictions when leaving home for work accounted for 18% of infections [8]. However, the proportion of participants using public transport (28% during restrictions, 48% post-Freedom Day) and visiting indoor hospitality (43%) and indoor leisure venues (41%) has increased with a corresponding increase in the proportion of infections attributable to indoor hospitality (APAF 7%) and leisure venues (APAF 10%), the use of which was largely restricted and transmissions prevented in the lockdown period [7]. As socialising behaviours, including eating and drinking in indoor restaurants, bars and clubs, using indoor gyms, attending the theatre, cinema and concerts, return to pre-pandemic levels, the relative contribution of these activities to transmission of SARS-CoV-2 is expected to grow.

In the absence of strict lockdowns and with the emergence of new highly transmissible variants with the ability to escape vaccines, policies which mitigate transmission in work settings, public transport and indoor hospitality and leisure venues will become more relevant. Recommendations for continued mask-wearing in vulnerable work settings, such as hospitals and care-homes, and improved indoor air filtration systems in high-footfall indoor venues are merited. Our work suggests that outdoor activities carry less risk and contributed to fewer infections than indoor activities, although our study was not powered to show a significant difference. Adequate investment to support hospitality and leisure venues to function outdoors, particularly in countries whose climate tends to be cold during seasonal peaks in SARS-COV-2 transmission, may have an important contribution to reducing transmission.

We found decreasing odds of infection with increasing frequency of shopping and hypothesise that doing a large once weekly shop might lead to more time being spent in a store with a greater potential for an increased number of unknown contacts than more frequent shorter shops in a smaller store with fewer people at any one time. This may account for the apparently paradoxical effect of an inverse relationship between shopping frequency and infection risk. We hypothesised that age is a strong determinant of contact patterns and that controlling for age would reduce or remove many of the associations found at the univariate level. However, with the exception of leaving home for work, the effect of age was less than anticipated. Similarly, while the risk of infection was associated with the number of close contacts outside the household (more than five contacts, OR 1.21 (1.01–1.45)), controlling for this, with the exception of leaving home for work, made little difference to associations with public transport and indoor hospitality and leisure activities. This suggests that these exposures are not mediated by recognised close contact and may represent more distant aerosol-based transmission [13]. This may also reflect an underreporting of close contacts due to difficulty remembering these, especially in public settings where the close contacts may not be known. The results of the model adjusting for non-household contacts should be interpreted with caution as it is possible that the number of contacts encountered is on the causal pathway (e.g. people avoid going to work because of the additional work contacts) and, although we did not observe a sizeable difference between the models adjusting for and not adjusting for number of contacts, the behavioural effects observed may be diluted.

### Strengths and limitations

The activities and behaviours are self-reported within the Virus Watch monthly surveys and therefore are subject to recall bias. We sought to minimise recall bias by asking about activities in the previous 7 days but it is possible that some recall bias remains. Additionally, adjustment for sociodemographic confounding is challenging and residual confounding may have affected findings. For example, recall bias and residual confounding may provide an alternative explanation for the greater association with less frequent shopping and the counter-intuitive protective effect of personal care activities (e.g. visiting a hairdresser). Although the activities were sampled at three points in the 5-month period between Freedom Day and mid-December, they may not be representative of time-varying activity levels throughout the period. We averaged data from three individual monthly behavioural surveys across the period to obtain an average measure which might be indicative of participant's general socialising behaviour as using one data-point per monthly survey may not be representative of behaviours for that specific month. This allowed for some expected variation in individual behaviour to give an overall estimate for the period. While having finer details of exposure behaviour, through for example, the use of data from weekly behavioural surveys which might directly precede symptom onset date, may have led to a better causal interpretation, we used the monthly data as this strengthened statistical power, as the number of infections during weekly periods was relatively low. Due to survey burden limitations our surveys did not capture information on usage of face coverings or other personal protective behaviours such as hand hygiene during specified exposures, either of which might act as a proxy for individual-level behaviours. Neither did we capture information on detailed features of the environment within which exposures took place, such as ventilation or screens. Our lack of ability to account for these factors may have influenced our results. We adjusted for vaccine status at entry to the study period but did not update vaccine status throughout the period which would provide a more accurate reflection of current infection risk. However, 94% of our cohort had been vaccinated at entry to the study period and the Omicron booster campaign had not yet started so this was unlikely to have influenced our results substantially. The Virus Watch surveys are subject to social desirability bias, however data examined during the first wave of the pandemic in Germany were found to support the use of self-reported contact survey data to reflect infection dynamics [14]. Virus Watch participants, who elect to be part of a research study examining associations between their activities and infection, may have had different frequency of exposure to activities than the general population. The Virus Watch cohort, and particularly those consistently answering the monthly activity surveys, has an underrepresentation of younger adults. We sought to minimise this bias by using an inverse probability weighting of the PAF estimates to the national age structure of the population in England and Wales. Both self-reported and linked data on test results from the national testing system allowed ascertainment of infections. Good ascertainment of SARS-COV-2 infections supports accurate assessment of the relative importance of risk factors.

## Conclusion

This research demonstrates that essential activities such as leaving home for work and public transport use continue to dominate infections but that indoor hospitality and indoor leisure activities increasingly contribute to infections. In times of widespread transmission of SARS-COV-2, increased population mixing as countries aim to ‘live with COVID’ and phasing out of mandatory isolation of positive cases, policies which mitigate transmission in work settings, public transport and indoor hospitality and leisure venues will become increasingly relevant.

## Data Availability

The raw data used in this study have been deposited in the ONS Secure Research Service. The data are available under restricted access as they contain sensitive health data. Access can be obtained by ONS Secure Research Service.

## Supplementary material

For supplementary material accompanying this paper visit http://doi.org/10.1017/S0950268822001832. click here to view supplementary material
