# Supplementary material for: Relative contribution of essential and non-essential activities to SARS-CoV-2 transmission following the lifting of public health restrictions in England and Wales
Source: Epidemiol Infect. 2022 Dec 7;151:e3. doi: 10.1017/S0950268822001832 (PMC9990391; doi:10.1017/S0950268822001832)
Supplement: Supplementary file 1 [file hygsup.zip › S0950268822001832sup001.docx]

Supplementary tables

Table S1. Infection by composite measures, unadjusted odds ratio, odds ratio adjusted for region, vaccine status, living alone, living with children, and living in a deprived area, adjusted odds ratios additionally adjusted for number of known contacts, and adjusted odds ratios additionally adjusted for age.

| **Activity** | **Category** | **N=11,413 (% in category)** | **Number of infections n= 493 (% within category)** | **Unadjusted OR (95% CI), p** | **Adjusted OR (95% CI), p**  **(n=11,232)** | **Adjusted OR additionally adjusted for number of contacts (95% CI), p** | **Adjusted OR additional adjusted for age (95% CI), p** |
| --- | --- | --- | --- | --- | --- | --- | --- |
| Leaves home for work or education | None  At least once | 7,294 (64%)  4,119 (36%) | 259 (3.6%)  234 (5.7%) | 1.00  1.64 (1.37 – 1.96)  p<0.0001 | 1.00  1.35 (1.11 – 1.64)  P=0.0029 | 1.00  1.34 (1.09 – 1.63)  P=0.0044 | 1.00  1.09 (0.88-1.33)  P=0.4354 |
| All public transport (no car share) | None  Any | 5,902 (52%)  5,511 (48%) | 219 (3.7%)  274 (4.9%) | 1.00  1.36 (1.13 – 1.62)  P=0.0009 | 1.00  1.27 (1.04 – 1.57)  P=0.0211 | 1.00  1.27 (1.03 – 1.56)  P=0.0236 | 1.00  1.27 (1.03 – 1.56)  P=0.0231 |
| Retail | None  Up to once  More than once to three times  More than three times | 482 (4%)  2,022 (18%)  5,745 (50%)  3,164 (28%) | 14 (2.9%)  110 (5.4%)  262 (4.6%)  107 (3.4%) | 0.85 (0.49 – 1.50)  1.64 (1.25 – 2.16)  1.37 (1.09 – 1.72)  1.00  P=0.0010 | 1.05 (0.58 – 1.88)  1.83 (1.36 – 2.45)  1.51 (1.19 – 1.92)  1.00  P=0.0001 | 1.06 (0.59 – 1.89)  1.83 (1.37 – 2.46)  1.52 (1.19 – 1.92)  1.00  P=0.0001 | 1.02 (0.57 – 1.84)  1.80 (1.34 – 2.42)  1.49 (1.18 – 1.90)  1.00  P=0.0002 |
| Indoor hospitality and socialising (pub, restaurant, party) | Up to once  More than once | 6,531 (57%)  4,882 (43%) | 258 (3.9%)  235 (4.8%) | 1.00  1.23 (1.03 – 1.47)  P=0.0254 | 1.00  1.21 (0.98 – 1.48)  P=0.0710 | 1.00  1.19 (0.98 – 1.47)  P=0.0853 | 1.00  1.21 (0.99 – 1.49)  P=0.0633 |
| Outdoor hospitality and socialising (pub, restaurant, party) | None  At least once | 6,777 (59%)  4,636 (41%) | 276 (4.1%)  217 (4.7%) | 1.00  1.16 (0.96 – 1.39)  P=0.1179 | 1.00  1.14 (0.94 – 1.39)  P=0.1860 | 1.00  1.14 (0.94 – 1.39)  P=0.1913 | 1.00  1.10 (0.91 – 1.34)  P=0.3275 |
| Indoor leisure (gym, theatre/cinema etc) | None  At least once | 6,713 (59%)  4,700 (41%) | 258 (3.8%)  235 (5.0%) | 1.00  1.32 (1.01 – 1.58)  P=0.0029 | 1.00  1.24 (1.02 – 1.51)  P=0.0284 | 1.00  1.24 (1.02 – 1.51)  P=0.0329 | 1.00  1.21 (0.99 – 1.48)  P=0.0531 |
| Outdoor leisure (team sport outdoors) | None  At least once | 10,570 (93%)  843 (7%) | 449 (4.3%)  44 (5.2%) | 1.00  1.24 (0.90 – 1.71)  p=0.1943 | 1.00  1.14 (0.82 – 1.59)  P=0.4521 | 1.00  1.13 (0.81 – 1.58)  P=0.4658 | 1.00  1.15 (0.83 – 1.61)  P=0.4130 |
| Non-social activity (hairdresser/beautician etc) | None  At least once | 7,469 (65%)  3,944 (35%) | 351 (4.7%)  142 (3.6%) | 1.00  0.76 (0.62 – 0.92)  P=0.0053 | 1.00  0.74 (0.59 – 0.90)  P=0.0031 | 1.00  0.73 (0.59 – 0.90)  P=0.0030 | 1.00  0.74 (0.60 – 0.91)  P=0.0037 |
| Region | East Midlands  East of England  London  North East  North West  South East  South West  Wales  W. Midlands  Yorkshire & The Humber  Missing | 1,065 (9%)  2,551 (23%)  1,202 (11%)  581 (5%)  1,192 (11%)  2,210 (19%)  924 (8%)  266 (2%)  626 (6%)  615 (5%)  181 | 35 (3.3%)  92 (3.6%)  57 (4.7%)  35 (6.0%)  62 (5.2%)  102 (4.6%)  33 (3.6%)  18 (6.8%)  33 (5.3%)  15 (2.4%) | 1.00  1.10 (0.74 – 1.64)  1.47 (0.95 – 2.25)  1.89 (1.17 – 3.05)  1.61 (1.06 – 2.46)  1.42 (0.96 – 2.11)  1.09 (0.67 – 1.77)  2.14 (1.19 – 3.83)  1.64 (1.01 – 2.66)  0.74 (0.39 – 1.36)  P=0.0028 | 1.00  1.12 (0.75 – 1.67)  1.04 (0.64 – 1.69)  1.89 (1.16 – 3.10)  1.58 (1.02 – 2.44)  1.47 (0.99 – 2.19)  1.20 (0.73 – 1.97)  2.61 (1.44 – 4.74)  1.53 (0.94 – 2.52)  0.69 (0.37 – 1.29)  P=0.0010 | 1.00  1.12 (0.75 – 1.67)  1.04 (0.64 – 1.69)  1.90 (1.16 – 3.11)  1.58 (1.02 -2.44)  1.47 (0.99 – 2.19)  1.20 (0.73 – 1.97)  2.62 (1.45 – 4.77)  1.54 (0.94 – 2.52)  0.69 (0.37 – 1.29)  P=0.0009 | 1.00  1.12 (0.75 – 1.68)  1.03 (0.64 – 1.68)  1.96 (1.19 – 3.20)  1.58 (1.02 – 2.44)  1.48 (0.99 – 2.22)  1.22 (0.74 – 1.99)  2.59 (1.43 – 4.72)  1.55 (0.95 – 2.54)  0.72 (0.89 – 1.34)  P=0.0010 |
| Vaccinated at study entry | Yes  No | 10,726 (94%)  687 (6%) | 464 (4.2%)  29 (4.3%) | 1.00  0.97 | 1.00  0.96 (0.64 – 1.43)  P=0.8360 | 1.00  0.96 (0.64 – 1.43)  P=0.8292 | 1.00  0.93 (0.62 – 1.39)  P=0.7219 |
| Lives alone | Lives alone  Lives with someone | 2,869 (25%)  8,544 (75%) | 81 (2.8%)  412 (4.8%) | 1.00  1.74 (1.37 – 2.22)  P <0.0001 | 1.00  1.54 (1.19 – 1.98)  P=0.0007 | 1.00  1.53 (1.19 – 1.98)  P=0.0007 | 1.00  1.54 (1.19 – 1.98)  P=0.0006 |
| Lives with children | No  Yes | 10,679 (94%)  734 (6%) | 404 (3.8%)  89 (12.1%) | 1.00  3.51 (2.58– 4.47)  p<0.0001 | 1.00  2.93 (2.25 – 3.82)  P<0.0001 | 1.00  2.93 (2.25 – 3.81)  P<0.0001 | 1.00  2.39 (1.82 – 3.13)  P<0.0001 |
| Residential area | Any rural  Any urban  Any conurbation  missing | 2,886 (26%)  5,209 (46%)  3,137 (28%)  181 | 106 (3.7%)  216 (4.2%)  160 (5.1%) | 1.00  1.13 (0.89 – 1.43)  1.41 (1.09 – 1.81)  P=0.0201 | 1.00  1.09 (0.86 – 1.39)  1.35 (0.99 – 1.84)  P=0.1508 | 1.00  1.09 (0.86 – 1.39)  1.35 (0.99 – 1.84)  P=0.1514 | 1.00  1.07 (0.84 – 1.37)  1.31 (0.96 – 1.78)  P=0.2148 |
| Deprivation score (IMD quintile) 1= most deprived | 1  2  3  4  5  Missing | 852 (8%)  1,589 (14%)  2,290 (20%)  3,032 (27%)  3,469 (31%)  181 | 44 (5.2%)  69 (4.3%)  94 (4.1%)  141 (4.7%)  134 (3.9%) | 1.36 (0.96 – 1.92)  1.13 (0.84 – 1.52)  1.07 (0.81 – 1.39)  1.21 (0.95 – 1.55)  1.00  P=0.3764 | 1.35 (0.94 – 1.95)  1.14 (0.84 – 1.56)  1.15 (0.88 – 1.52)  1.24 (0.97 – 1.58)  1.00  P=0.3956 | 1.35 (0.94 – 1.96)  1.14 (0.84 – 1.56)  1.16 (0.88 – 1.52)  1.24 (0.97 – 1.59)  1.00  P=0.3876 | 1.27 (0.88 – 1.83)  1.08 (0.79 – 1.47)  1.13 (0.85 – 1.49)  1.21 (0.94 – 1.55)  1.00  P=0.5570 |
| Age | Working Age  65 and above  Missing | 5,370 (47%)  6,042 (53%)  1 | 331 (6.2%)  162 (2.7%) | 2.38 (1.97 – 2.89)  1.00  P<0.0001 | Not in model | Not in model | 1.87 (1.50 – 2.34)  1.00  P<0.000* |
| Contacts | 0 – 5  More than 5 | 6,124 (54%)  5,289 (46%) | 242 (3.9%)  251 (4.8%) | 1.00  1.21 (1.01- 1.45)  P=0.0377 | Not in model | 1.00  1.05 (0.86 – 1.28)  P=0.5101 | Not in model |

*wald test value given when there was missing data on the co-variable
