# Supplementary material for: Relative contribution of essential and non-essential activities to SARS-CoV-2 transmission following the lifting of public health restrictions in England and Wales
Source: Epidemiol Infect. 2022 Dec 7;151:e3. doi: 10.1017/S0950268822001832 (PMC9990391; doi:10.1017/S0950268822001832)
Supplement: Supplementary file 1 [file hygsup.zip › S0950268822001832sup002.docx]

Table S2 Infection according to transport methods, unadjusted odds ratios and odds ratios adjusted for region, vaccination status, living alone, living with children, living in a deprived area

| **Characteristic** | **Category** | **N=11,413(% in category)** | **Number of infections n= 493 (% within category)** | **Unadjusted OR** | **Adjusted OR, 95% CI, p (n=11,232)** |
| --- | --- | --- | --- | --- | --- |
| Taxi | None  At least once | 9,217 (81%)  2,196 (19%) | 376 (4.1%)  117 (5.3%) | 1.00  1.32 (1.07 – 1.64)  P=0.0117 | 1.00  1.28 (1.03 – 1.59)  P=0.0321 |
| Shared car | None  At least once | 4,634 (41%)  6,779 (59%) | 211 (4.6%)  282 (4.2%) | 1.00  0.91(0.76 – 1.09)  P=0.3112 | 1.00  0.99 (0.83 – 1.20)  P=0.9642 |
| Bus | None  At least once | 8,250 (72%)  3,163 (28%) | 342 (4.2%)  151 (4.8%) | 1.00  1.16 (0.95 – 1.41)  P=0.1432 | 1.00  1.27 (1.03 – 1.56)  P=0.0291 |
| Overground train or tram | None  At least once | 8,694 (76%)  2,719 (24%) | 344 (3.9%)  149 (5.5%) | 1.00  1.41 (1.16 – 1.71)  P=0.0009 | 1.00  1.41 (1.14 – 1.74)  P=0.0017 |
| Underground train | None  At least once | 9,709 (85%)  1,704 (15%) | 409 (4.2%)  84 (4.9%) | 1.00  1.18 (0.93 – 1.49)  P=0.1870 | 1.00  1.14 (0.87 – 1.51)  P=0.3406 |
| Airplane | None  At least once | 10,338 (91%)  1,075 (9%) | 443 (4.3%)  50 (4.7%) | 1.00  1.09 (0.81 – 1.47)  P=0.5783 | 1.00  1.04 (0.76 – 1.41)  P=0.8259 |
