# Supplementary material for: Relative contribution of essential and non-essential activities to SARS-CoV-2 transmission following the lifting of public health restrictions in England and Wales
Source: Epidemiol Infect. 2022 Dec 7;151:e3. doi: 10.1017/S0950268822001832 (PMC9990391; doi:10.1017/S0950268822001832)
Supplement: Supplementary file 1 [file hygsup.zip › S0950268822001832sup003.docx]

Table S3 Infection by individual activities, unadjusted odds ratios and odds ratios adjusted for region, vaccination status, living alone, living with children, living in a deprived area

| **Activity** | **Frequency (number of times in a week)** | **N=11,413(% in category)** | **Number of infections n=493(% within category)** | **Unadjusted OR (95% CI), p** | **Adjusted OR, (95% CI), p (n=11,232)** |
| --- | --- | --- | --- | --- | --- |
| Essential shopping | Up to once  More than once to twice  More than twice | 3,756 (33%)  3,877 (34%)  3,780 (33%) | 182 (4.9%)  180 (4.6%)  131 (3.5%) | 1.00  0.96 (0.77 – 1.18)  0.71 (0.56 – 0.87)  P=0.0052 | 1.00  1.02 (0.82 – 1.26)  0.72 (0.57 – 0.92)  P=0.0072 |
| Non-essential shopping | None  Up to once  More than once | 4,073 (36%)  5,224 (46%)  2,116 (19%) | 183 (4.5%)  222 (4.3%)  88 (4.2%) | 1.00  0.94 (0.77 – 1.15)  0.92 (0.71 – 1.19)  P=0.7832 | 1.00  0.93 (0.76 – 1.14)  0.89 (0.68 – 1.16)  P=0.6399 |
| Indoor pub, bar, club | None  Up to once  More than once | 6,137 (54%)  3,373 (29%)  1,903 (17%) | 243 (3.9%)  147 (4.4%)  103 (5.4%) | 1.00  1.11(0.89 – 1.36)  1.39 (1.09 – 1.76)  P=0.0288 | 1.00  1.14 (0.92 – 1.40)  1.45 (1.13 – 1.84)  P=0.0138 |
| Outdoor pub, bar, club | None  At least once in 3 months | 9,088 (80%)  2,325 (20%) | 387 (4.3%)  106 (4.6%) | 1.00  1.07 (0.86 – 1.34)  P=0.5271 | 1.00  1.09 (0.87 – 1.36)  P=0.4653 |
| Indoor restaurant, café, canteen | None  Up to once  More than once | 3,325 (29%)  5,320 (47%)  2,768 (24%) | 119 (3.6%)  263(4.9%)  111 (4.0%) | 1.00  1.40 (1.12 – 1.75)  1.13 (0.86 – 1.47)  P=0.0063 | 1.00  1.38 (1.11 – 1.73)  1.14 (0.87 – 1.49)  P=0.0120 |
| Outdoor restaurant, café, canteen | None  At least once in 3 months | 8,147 (71%)  3,266 (29%) | 342 (4.2%)  151 (4.6%) | 1.00  1.11(0.91 – 1.35)  P=0.3153 | 1.00  1.14 (0.94 – 1.39)  P=0.1939 |
| Indoor party | None  At least once in 3 months | 9,967 (87%)  1,446 (13%) | 408 (4.1%)  85 (5.9%) | 1.00  1.46 (1.15 – 1.86)  P=0.0028 | 1.00  1.38 (1.08 – 1.77)  P=0.0124 |
| Outdoor party | None  At least once in 3 months | 10,959 (96%)  454 (4%) | 474 (4.3%)  19 (4.2%) | 1.00  0.97 (0.60 – 1.54)  P=0.8850 | 1.00  0.91 (0.55 – 1.49)  P=0.7110 |
| Gym/indoor sport | None  At least once in 3 months | 8,894 (78%)  2,519 (22%) | 361 (4.1%)  132 (5.2%) | 1.00  1.31(1.07 – 1.60)  P=0.0118 | 1.00  1.32 (1.07 – 1.63)  P=0.0105 |
| Team sport outdoors | None  At least once in 3 months | 10,570 (93%)  843 (7%) | 449 (4.3%)  44 (5.2%) | 1.00  1.24 (0.90 – 1.71)  P=0.1943 | 1.00  1.23 (0.89 – 1.71)  P=0.2269 |
| Theatre, cinema, concert, sports event | None  At least once in 3 months | 8,250 (72%)  3,163 (28%) | 336 (4.1%)  157 (4.9%) | 1.00  1.23 (1.01 – 1.49)  P=0.0387 | 1.00  1.23 (1.01 – 1.49)  P=0.0464 |
| Hairdresser, barber, beautician | None  At least once in 3 months | 7,469 (65%)  3,944 (35%) | 351 (4.7%)  142 (3.6%) | 1.00  0.76 (0.62 – 0.92)  P=0.0053 | 1.00  0.76 (0.62 – 0.93)  P=0.0078 |
