# Supplementary material for: Relative contribution of essential and non-essential activities to SARS-CoV-2 transmission following the lifting of public health restrictions in England and Wales
Source: Epidemiol Infect. 2022 Dec 7;151:e3. doi: 10.1017/S0950268822001832 (PMC9990391; doi:10.1017/S0950268822001832)
Supplement: Supplementary file 1 [file hygsup.zip › S0950268822001832sup004.docx]

Table S4 Infection by composite measures, unadjusted odds ratios and odds ratios adjusted for covariables as proposed by alternative DAG: region, living alone, living with children, living in a deprived area, and ethnicity, adjusted odds ratios additionally adjusted for number of known contacts, adjusted odds ratios additionally adjusted for age

| **Activity** | **Category** | **N=11,413 (% in category)** | **Number of infections n= 493 (% within category)** | **Unadjusted OR (95% CI), p** | **Adjusted OR (95% CI), p** | **Adjusted OR additionally adjusted for number of known contacts (95% CI), p** | **Adjusted OR additionally adjusted for age (95% CI), p** |
| --- | --- | --- | --- | --- | --- | --- | --- |
| Leaves home for work or education | None  At least once | 7,294 (64%)  4,119 (36%) | 259 (3.6%)  234 (5.7%) | 1.00  1.64 (1.37 – 1.96)  p<0.0001 | 1.00  1.34 (1.09 – 1.62)  P=0.0038 | 1.00  1.32 (1.09 – 1.62)  P=0.0058 | 1.00  1.08 (0.88-1.33)  P=0.4631 |
| Public transport | None  At least once | 5,902 (52%)  5,511 (48%) | 219 (3.7%)  274 (4.9%) | 1.00  1.36 (1.13 – 1.62)  P=0.0009 | 1.00  1.28 (1.04 – 1.57)  P=0.0206 | 1.00  1.27 (1.03 – 1.57)  P=0.0232 | 1.00  1.28 (1.04 – 1.57)  P=0.0214 |
| Retail | None  Up to once  More than once to thrice  More than thrice | 482 (4%)  2,022 (18%)  5,745 (50%)  3,164 (28%) | 14 (2.9%)  110 (5.4%)  262 (4.6%)  107 (3.4%) | 0.85 (0.49 – 1.50)  1.64 (1.25 – 2.16)  1.37 (1.09 – 1.72)  1.00  P=0.0010 | 1.06 (0.59 – 1.89)  1.83 (1.37 – 2.46)  1.51 (1.19 – 1.92)  1.00  P=0.0002 | 1.06 (0.59 – 1.91)  1.84 (1.37 – 2.47)  1.51 (1.19 – 1.92)  1.00  P=0.0002 | 1.03 (0.57 – 1.85)  1.81 (1.35 – 2.43)  1.49 (1.17 – 1.89)  1.00  P=0.0002 |
| Indoor hospitality and socialising (pub, restaurant, party) | Up to once  More than once | 6,531 (57%)  4,882 (43%) | 258 (3.9%)  235 (4.8%) | 1.00  1.23 (1.03 – 1.47)  P=0.0254 | 1.00  1.21 (0.99 – 1.48)  P=0.0661 | 1.00  1.20 (0.98 – 1.47)  P=0.0804 | 1.00  1.22 (0.99 – 1.49)  P=0.0605 |
| Outdoor hospitality and socialising (pub, restaurant, party) | None  At least once | 6,777 (59%)  4,636 (41%) | 276 (4.1%)  217 (4.7%) | 1.00  1.16 (0.96 – 1.39)  P=0.1179 | 1.00  1.14 (0.94 – 1.39)  P=0.1840 | 1.00  1.14 (0.94 – 1.39)  P=0.1894 | 1.00  1.10 (0.91 – 1.34)  P=0.3278 |
| Indoor leisure (gym, theatre/cinema etc) | None  At least once | 6,713 (59%)  4,700 (41%) | 258 (3.8%)  235 (5.0%) | 1.00  1.32 (1.01 – 1.58)  P=0.0029 | 1.00  1.24 (1.02 – 1.51)  P=0.0312 | 1.00  1.23 (1.01 – 1.50)  P=0.0362 | 1.00  1.21 (0.99 – 1.47)  P=0.0571 |
| Outdoor leisure (team sport outdoors) | None  At least once | 10,570 (93%)  843 (7%) | 449 (4.3%)  44 (5.2%) | 1.00  1.24 (0.90 – 1.71)  p=0.1943 | 1.00  1.14 (0.81 – 1.58)  P=0.4605 | 1.00  1.13 (0.81 – 1.58)  P=0.4755 | 1.00  1.15 (0.82 – 1.60)  P=0.4224 |
| Non-social activity (hairdresser/beautician etc) | None  At least once | 7,469 (65%)  3,944 (35%) | 351 (4.7%)  142 (3.6%) | 1.00  0.76 (0.62 – 0.92)  P=0.0053 | 1.00  0.74 (0.60 – 0.91)  P=0.0039 | 1.00  0.74 (0.60 – 0.91)  P=0.0038 | 1.00  0.74 (0.60 – 0.91)  P=0.0044 |
| Region | East Midlands  East of England  London  North East  North West  South East  South West  Wales  W. Midlands  Yorkshire & The Humber  Missing | 1,065 (9%)  2,551 (23%)  1,202 (11%)  581 (5%)  1,192 (11%)  2,210 (19%)  924 (8%)  266 (2%)  626 (6%)  615 (5%)  181 | 35 (3.3%)  92 (3.6%)  57 (4.7%)  35 (6.0%)  62 (5.2%)  102 (4.6%)  33 (3.6%)  18 (6.8%)  33 (5.3%)  15 (2.4%) | 1.00  1.10 (0.74 – 1.64)  1.47 (0.95 – 2.25)  1.89 (1.17 – 3.05)  1.61 (1.06 – 2.46)  1.42 (0.96 – 2.11)  1.09 (0.67 – 1.77)  2.14 (1.19 – 3.83)  1.64 (1.01 – 2.66)  0.74 (0.39 – 1.36)  P=0.0028 | 1.00  1.12 (0.75 – 1.68)  1.07 (0.66 – 1.76)  1.88 (1.15 – 3.07)  1.59 (1.03 – 2.45)  1.46 (0.98 – 2.18)  1.21 (0.74 – 1.97)  2.58 (1.42 – 4.69)  1.56 (0.95 – 2.56)  0.71 (0.38 – 1.32)  P=0.0016 | 1.00  1.12 (0.75 – 1.68)  1.07 (0.66 – 1.76)  1.88 (1.15 – 3.08)  1.59 (1.03 - 2.46)  1.46 (0.98 – 2.18)  1.20 (0.73 – 1.96)  2.59 (1.43 – 4.71)  1.56 (0.95 – 2.57)  0.71 (0.38 – 1.32)  P=0.0016 | 1.00  1.13 (0.75 – 1.68)  1.08 (0.66 – 1.77)  1.94 (1.18 – 3.17)  1.59 (1.03 – 2.46)  1.48 (0.99 – 2.21)  1.22 (0.74 – 1.99)  2.57 (1.41 – 4.67)  1.58 (0.96 – 2.59)  0.73 (0.39 – 1.35)  P=0.0017 |
| Ethnic group | White British  White Other  Asian  Black  Mixed  Other  Missing | 10,286 (91%)  628 (6%)  213 (2%)  54 (<1%)  91 (1%)  49 (<1%)  92 | 433 (4.2%)  31 (4.9%)  9 (4.2%)  5 (9.3%)  8 (8.8%)  1 (2.0%) | 1.00  1.18 (0.81 – 1.72)  1.00 (0.51 – 1.97)  2.32 (0.92 – 5.86)  2.19 (1.05 – 4.56)  0.47 (0.07 – 3.44)  P=0.1882 | 1.00  0.93 (0.62 – 1.39), p=0.735  0.69 (0.33 – 1.47), p=0.343  1.75 (0.66 – 4.66), p=0.263  1.69 (0.79 – 3.63), p=0.178  0.33 (0.04 – 2.49), p=0.284  * | 1.00  0.93 (0.62 – 1.39), p=0.725  0.69 (0.33 – 1.47), p=0.343  1.75 (0.66 – 4.66), p=0.261  1.69 (0.79=8 – 3.63), p=0.180  0.33 (0.04 – 2.49), p=0.285 | 1.00  0.88 (0.59 – 1.32), p=0.551  0.66 (0.31 – 1.39), p=0.272  1.64 (0.62 – 4.34), p=0.324  1.58 (0.73 – 3.38), p=0.243  0.32 (0.04 – 2.39), p=0.269 |
| Lives alone | Lives alone  Lives with someone | 2,869 (25%)  8,544 (75%) | 81 (2.8%)  412 (4.8%) | 1.00  1.74 (1.37 – 2.22)  P <0.0001 | 1.00  1.55 (1.19 – 1.99)  P=0.0005 | 1.00  1.54 (1.19 – 1.99)  P=0.0006 | 1.00  1.55 (1.19 – 1.99)  P=0.0005 |
| Lives with children | No  Yes | 10,679 (94%)  734 (6%) | 404 (3.8%)  89 (12.1%) | 1.00  3.51 (2.58– 4.47)  p<0.0001 | 1.00  2.96 (2.27 – 3.87)  P<0.0001 | 1.00  2.96 (2.27 – 3.88)  P<0.0001 | 1.00  2.43 (1.85 – 3.18)  P<0.0001 |
| Residential area | Any rural  Any urban  Any conurbation  Missing | 2,886 (26%)  5,209 (46%)  3,137 (28%)  181 | 106 (3.7%)  216 (4.2%)  160 (5.1%) | 1.00  1.13 (0.89 – 1.43)  1.41 (1.09 – 1.81)  P=0.0201 | 1.00  1.09 (0.85 – 1.39)  1.35 (0.99 – 1.84)  P=0.1567 | 1.00  1.09 (0.85 – 1.39)  1.35 (0.99 – 1.84)  P=0.1571 | 1.00  1.06 (0.83 – 1.36)  1.31 (0.96 – 1.78)  P=0.2129 |
| Deprivation score (IMD quintile) 1= most deprived | 1  2  3  4  5  Missing | 852 (8%)  1,589 (14%)  2,290 (20%)  3,032 (27%)  3,469 (31%)  181 | 44 (5.2%)  69 (4.3%)  94 (4.1%)  141 (4.7%)  134 (3.9%) | 1.36 (0.96 – 1.92)  1.13 (0.84 – 1.52)  1.07 (0.81 – 1.39)  1.21 (0.95 – 1.55)  1.00  P=0.3764 | 1.34 (0.93 – 1.94)  1.14 (0.84 – 1.56)  1.16 (0.88 – 1.53)  1.22 (0.96 – 1.57)  1.00  P=0.4425 | 1.34 (0.93 – 1.95)  1.14 (0.84 – 1.56)  1.16 (0.88 – 1.53)  1.23 (0.96 – 1.57)  1.00  P=0.4334 | 1.26 (0.87 – 1.83)  1.09 (0.79 – 1.48)  1.13 (0.86 – 1.49)  1.19 (0.93 – 1.53)  1.00  P=0.6045 |
| Age | Working Age  65 and above  Missing | 5,370 (47%)  6,042 (53%)  1 | 331 (6.2%)  162 (2.7%) | 2.38 (1.97 – 2.89)  1.00  P<0.0001 | Not in model | Not in model | 1.87 (1.49 – 2.33)  1.00  P<0.001* |
| Contacts | 0 – 5  More than 5 | 6,124 (54%)  5,289 (46%) | 242 (3.9%)  251 (4.8%) | 1.00  1.21 (1.01- 1.45)  P=0.0377 | Not in model | 1.00  1.05 (0.86 – 1.28)  P=0.6341 | Not in model |

***where there are missing values, Wald test p-values are given**
